# Recreational fisheries economics between illusion and reality: The case of Algeria

**DOI:** 10.1371/journal.pone.0201602

**Published:** 2018-08-02

**Authors:** Nadhéra Babali, Mohamed Kacher, Dyhia Belhabib, Ferial Louanchi, Daniel Pauly

**Affiliations:** 1 National Research Center for the Development of Fisheries and Aquaculture (CNRDPA), Boulevard front de Mer, Bou Ismail, Tipaza, Algeria; 2 National School of Marine Science and Coastal Management (ENSSMAL), Campus Universitaire Dely Ibrahim, Bois des Cars, Algiers, Algeria; 3 Ministry of Agriculture, Rural Development and Fisheries (MADRP), Algiers, Algeria; 4 Sea Around Us, University of British Columbia, Vancouver, BC, Canada; Aristotle University of Thessaloniki, GREECE

## Abstract

Recreational fishing is often perceived as harmless when it comes to fisheries management, and its impact often estimated to surpass the economic outcomes of e.g. large-scale fisheries. Recreational fisheries are often an indication of political stability and sound ecosystem management. However, despite a high economic impact, the economic costs on traditional and small-scale commercial fishers is yet to be known. This paper answers the question of how unregulated recreational fisheries could rather generate a loss to an economy, and cause unfair competition with existing commercial sectors using the example of Algeria. This paper assesses catches and economic value of recreational fisheries in Algeria, and finds that over 6,000 tonnes reach commercial markets annually, competing directly with the small-scale artisanal sector, while selling recreationally caught fish is still illegal. The paper further finds that the public is thereby deprived—through lost tax, licence income and landed value of $45 million US annually.

## Introduction

Fishing is one of the oldest activities on earth [1]. With fisheries industrialization, fish stocks are increasingly subject to over-exploitation and are declining at an alarming rate [2, 3]. This over-exploitation is further exacerbated by the impacts of climate change, which drive fish migrations and local depletions in some of the poorest areas of the world [4]. In addition, illegal, Unregulated and Unreported fishing by industrial fleets, often associated with over-exploitation of fish stocks, and other environmental crimes, is considered one of the major threats to fisheries, today [5]. However, other sectors whose impact is often unknown also contribute to this trend. Indeed, beyond the fact that recreational catches are often under-reported, the sector remains mainly unregulated in many countries of the world [6]. Catch reconstructions revealed that data reported to the United Nations’ Food and Agriculture Organization (FAO) do not reflect this reality [7]. Thus, in The Bahamas for example, previously unaccounted recreational catches by tourists were estimated at around 662,000 t per year (75% of the total annual commercial catch) [8].

**Fig 1:**
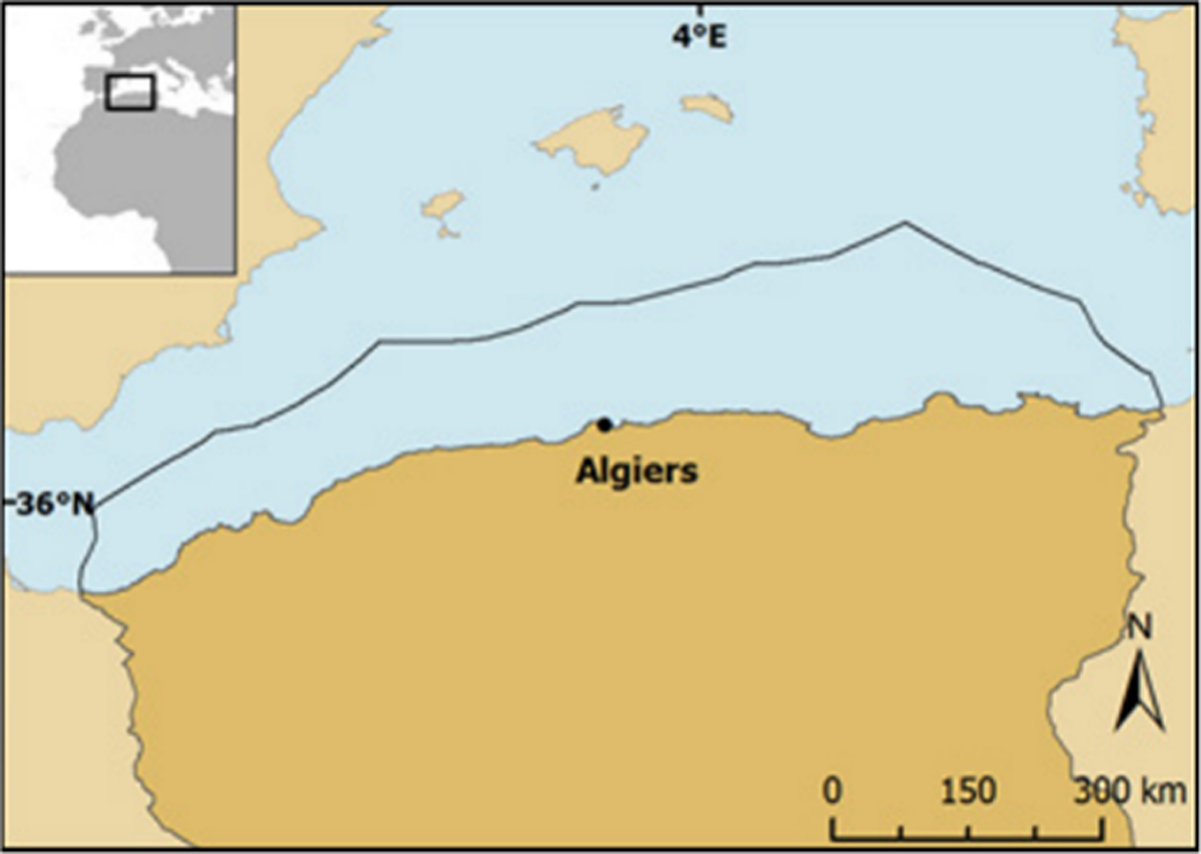
Map of the reserved fishing zone of Algeria.

In Algeria, the largest country of Africa (Fig 1), the recreational fishing sector is not regulated and its catches remain unmonitored. During a previous study, it was estimated that catches by the small-scale sector reached over 83,000t of which 1,000 t were generated by recreational fishing in 2010, which were completely unreported [9]. Algerian fisheries are operated by relatively small vessels within the Mediterranean due to sociological and geomorphologic attributes documented in [9]. The Algerian Fisheries Acts define artisanal fishing as “*any traditional commercial fishing practiced in territorial sea”* and the coastal fishing as *“*Any traditional practice of commercial fishing close to coast [10], they allow the largest variety of gears compared to others fishing categories and limit the length of small scale fishing vessels at a maximum of 12 meter [11]. Given the employment and the decade long security crisis in the country–mainly triggered by the lack of income opportunities and a social dissatisfaction by the youth [12], the Algerian Government uses the fishing sector as an alternative source of income, and heavily subsidizes small scale fishing vessels “to create employment opportunities”[13, 14].

In the Algerian regulation, Recreational fisheries include “*on-foot fishing; fishing on board of recreational vessels; spearfishing”*[15]. It is defined as: “*Any fishing exercise as a sport or as a leisure activity and for non-commercial purposes*” [10].

Referring to the definition above, the fact that Recreational catches cannot be sold assumes no tax revenue from catches to the government and that the recreational sector should not compete with the commercial sector. To practice recreational fishing, fishers must have a recreational fishing authorizations (License) issued by local fisheries authorities after payment of an annual fee and validation of the applicant's file by a local commission in charge of granting licenses and their renewal. Boat fishers have to provide vessel characteristics to authorities, while spearfishers must have a weapon license. Despite the fact that the recreational sector is not well developed in Algeria, thanks to a decade long security crisis along the coast, increasing evidence of conflict with the commercial sector, suggests its economic footprint is much larger than what is suggested in previous reports [16]. This paper assesses the catch and economic footprint for recreational fisheries, and the potential for conflict with the artisanal commercial sector as illustrated through the case of Algeria, and discusses gaps in regulations and policy within the Algerian context.

## Methods

### Recreational vessel catches

Recreational catches for 14 coastal *wilayas* (districts, with particularities relating to the national legislation) of Algeria are either not reported (most of the time) or are reported as part of the commercial catch. We estimate these catches based on annual Catch Per Unit of Effort (CPUE) data, where effort is defined as the number of actives vessels. We note that the size of the vessels, their geographic fishing range, and fishing activities are assumed homogeneous, and hence, we consider this as an appropriate measure of the fishing effort.

Initial- data on artisanal commercial vessel (small-scale professional) catches recorded daily by observers were obtained from one of Western Algeria’s landing sites and analyzed. Vessel name and registration number revealed that, from January 2012 to September 2014, reported landings of small-scale professional fisheries also included catches of recreational vessels (locales fisheries authorities, unpub. data). These data were reported to the FAO, which does not distinguish between catch types [7]. However the question remains raised with regards to how much of the remaining catch is not reported, and what is its economic footprint.

Daily catch and effort data for 2012 were available, which allowed to estimate the CPUE for that year. The number of fishing trips per year depends on seasons, varying from 28 (vessels fishing only in summer) to 116 (vessels fishing throughout the year). This information was available for 9 recreational vessels. The landings of each vessel were reported per day, per species, genus, or family and ‘per box’ and not by weight (locales fisheries authorities, unpub. data, S1 Table). Each box is equivalent to 17 kg for pelagic fish and 14 kg for demersal fish in wet weight. We estimated the annual landing for each vessel by adding up the daily landings of all species combined. Hence, we estimated an average CPUE. we then extrapolated harbor-wide using the total number of recreational vessels, i.e. 9 vessels.

Finally, to estimate the coast-wide catch, we first obtained the number of recreational fishing licenses (one license per vessel) from the relevant fisheries departments of the 14 coastal *wilayas*, then, assuming an even distribution of landings along the Algerian coast [17, 18, 19] we multiplied the average CPUE by the number of licensed recreational vessels.

We assume here that all application for license is intended to a commercial fishing, because fishing for subsistence doesn't require any license [20]. There is no known threshold of how much of the recreational catch is sold, however, professional fishers spoken with during the course of this study agreed that at least "most" of the catch is sold in local markets, which constitutes an unfair competition. Hence, herein, we conservatively assume that 75% of the catch is sold in local markets.

### Spearfishing catches

To estimate recreational spearfishing catches, we multiplied the number of individual licenses obtained from the *wilayas’* fisheries register, where one fisher gets one license, by the number of fishing days, assessed as 50 days·fisher^-1^·year^-1^, and the average daily weight of 20 kg·fisher^-1^·day^-1^ from [20]. We assumed both the CPUE and the number of fishing days remained constant over the 2011–2015 time period.

### Fishers’ income from recreational fisheries and government lost revenue

The Algerian government collects taxes from all commercial sectors, and given the prohibition of trade of recreationally caught fish, the Government does not collect any taxes from recreational fishers, assuming fishers abide by the law. Herein, we calculated the potential economic loss to the Government that is attributable to this unregulated fishery as the difference between the revenue that the license fees and other fees like social security, crew role, … etc would have brought in if considered artisanal (1,200 USD per vessel) and the license fees that they bring as they are considered recreational (30 USD) [20], knowing that most recreational catch is sold in local markets (pers. observation.). The difference represents a nontaxable income to local recreational fishers.

Hence, the total revenue earned by recreational fishers is calculated as the discount on the cost of the license and other fees, i.e. the difference between the cost of a full commercial license (1,200USD)–noting that vessel size is similar, and geographic fishing range, and fishing capacity are similar–and that of a recreational license (30 USD), plus the revenue from the catch itself (or landed value).

The operating costs (fuel and lubricant) are deduced from the total revenue, on the basis of an average number of trips of 73 days per year multiplied by a cost estimated at 2,300 DA by trip, according to small scale fishers with whom we conversed.

Hence, the total operating cost is estimated as the cost per day, multiplied by the number of trips (fishing days), multiplied by the number of licenses (which reflects the number of boats, as one boat gets one license). Therefore, profits from the recreational sector gained by fishers, are estimated as the total revenue i.e. average ex-cessel price multiplied by the catch per year, plus the value they would otherwise pay if they were considered professional (i.e. lost to government), minus the operating costs.

The ex-vessel price is estimated at 675 AD (Algerian Dinar) in 2015 ($1 US = 100 AD) as extracted from the Algerian National Fisheries Database (Government of Algeria, unpub. data).

## Results

### Recreational vessels catch

**Fig 2:**
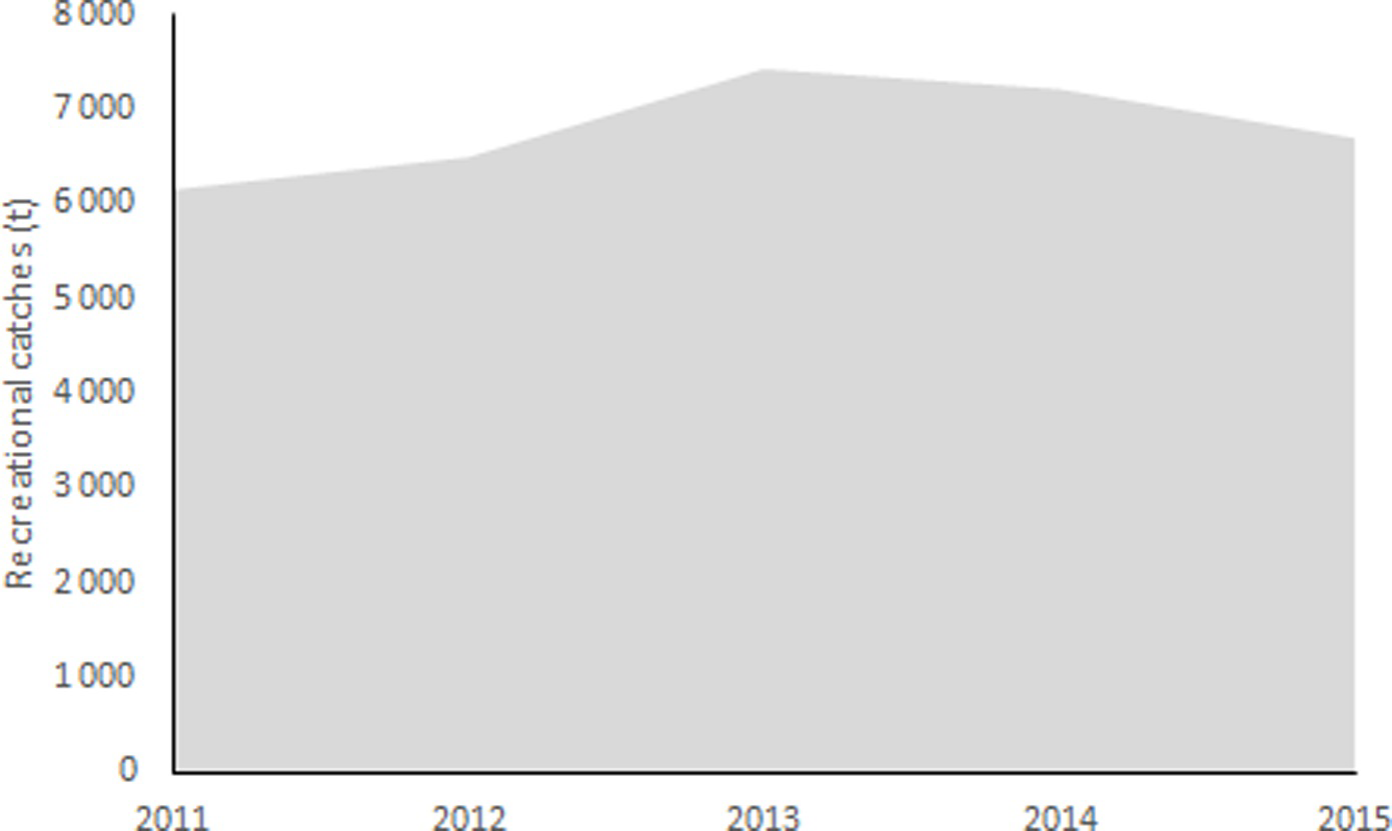
Reconstructed recreational catches of Algeria, 2011–2015.

The recreational landing is estimated at 3, 256.28 kg·vessel^-1^·year^-1^.The reconstructed catch shows a slight increase from 5,529 tonnes in 2011 to around 6,730 tonnes in 2013, and decreased slightly to less than 6,000 t in 2015 (Table 1 and Fig 2). We note that, 47% of the vessels at the sample landing port used as a geographic anchor point herein are recreational. Assuming catch data for each landing site is fully reported–which is not the case–a considerable portion of the small-scale catch data would be originating from the recreational sector.

**Table 1:** Number of authorizations and CPUE used to calculate recreational catches in Algeria, 2011–2015.

Years	Number of licenses	Average CPUE (kg·vessel^-1^)	Recreational landings (tonnes)
**2011**	1,698	3,325	5,646
**2012**	1,780	3,325	5,919
**2013**	2,067	3,325	6,873
**2014**	1,987	3,325	6,607
**2015**	1,832	3,325	6,092

**Fig 3:**
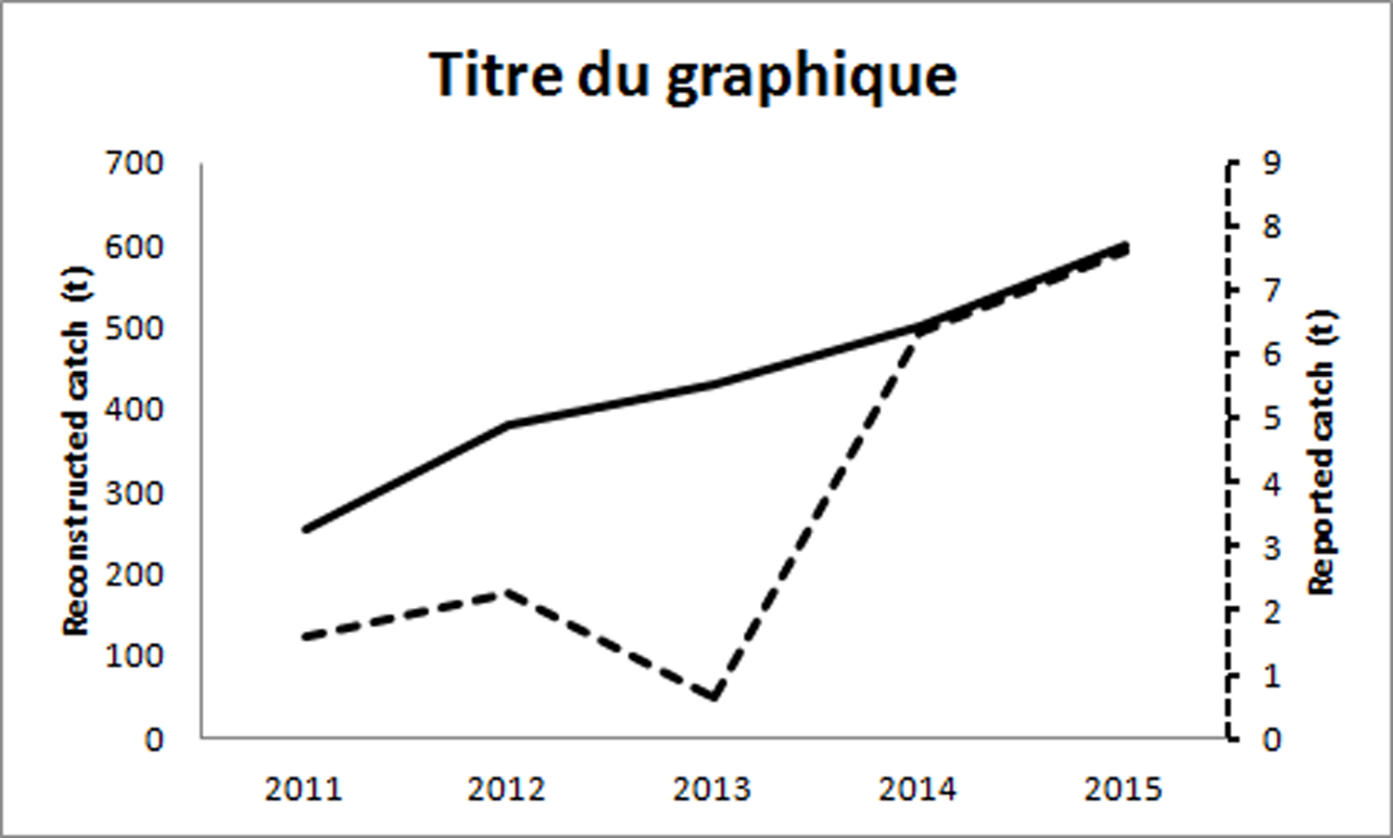
Comparison between the official data (dashed line) and the reconstructed catch (hard line) in the *wilaya* of Boumerdes, Algeria.

Overall, our estimate is 6 times higher than the conservative estimate of [9] comparing the first year for which we have data, i.e. 2011 to that estimate for the year 2010. Given the lack of information on reporting nationally, we use the example of one *wilaya* to compare reported landings to unreported catches. In the *wilaya* of Boumerdes, officially reported catches from 2011 and 2015 range from 1.6 t to 7.5 t annually [21], while the reconstructed catch varies between 253 t to 599 t during the same period (Fig 3), illustrating a high disparity and major under-reporting of recreational catches.

Catches by recreational fishing vessels include 21% of sparids, 10% sardinellas (*Sardinelaaurita*), 10% bogue (*Boopsboops*), 8.3% horse mackerel (*Trachurus* spp.), 0.03% swordfish (*Xiphiasgladius*) along with other tunas and billfishes (around 6%), 19% cephalopods, 1% hake (*Merlucciusmerluccius*); 0.6% sardine (*Sardinapilchardus*), the remainder consisting of other fishes (Soleidae, Mugilidae, Triglidae, Rajidae, Serranidae, etc.) and overlapping with the species targeted by the small-scale commercial sector.

### Spearfishing catches

**Fig 4:**
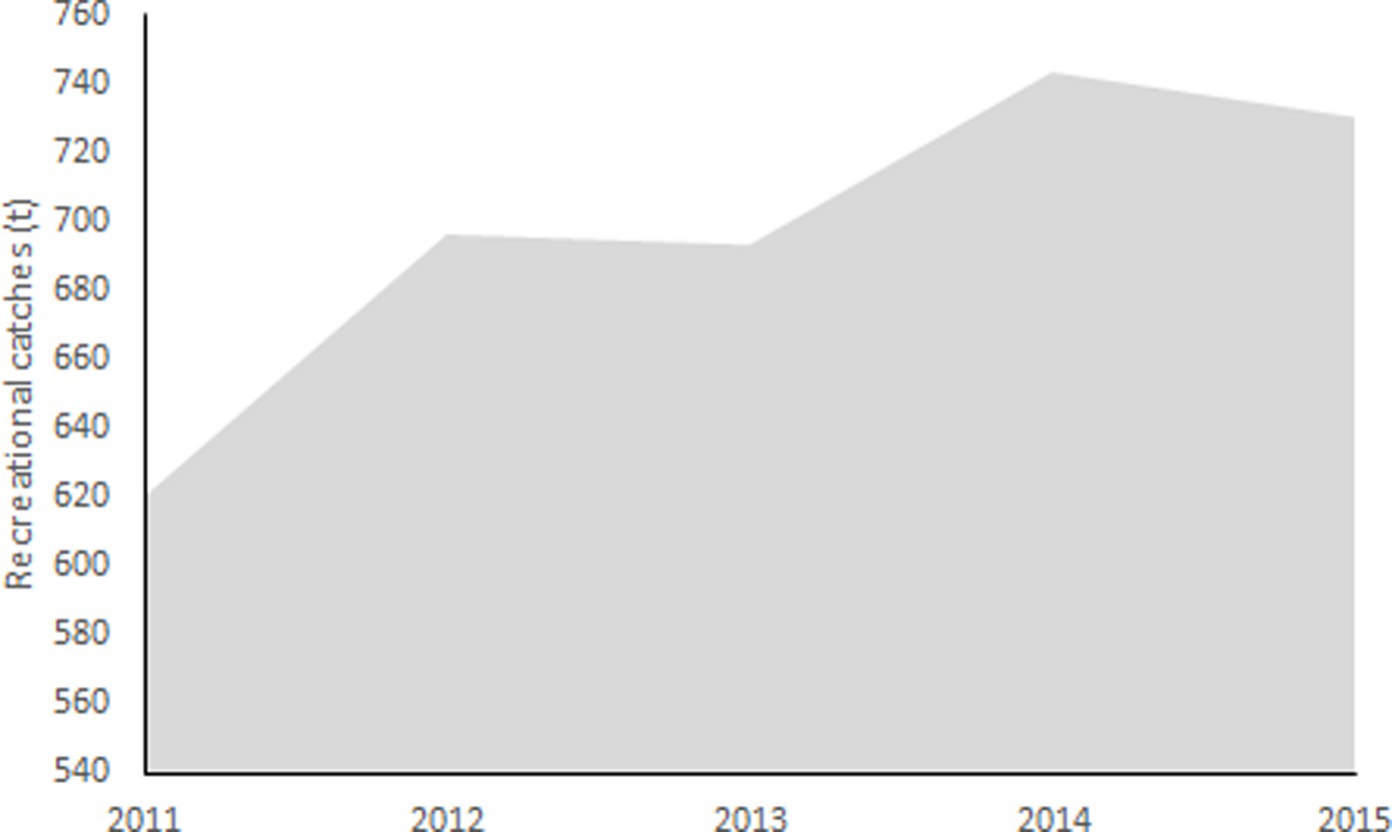
Reconstructed recreational catches from the spearfishing sub-sector in Algeria, 2011–2015.

Spearfishing catches estimated increased from 620 tonnes in 2011 to 730 tonnes in 2015 (Table 2 and Fig 4). The composition of the spearfished catch depends on the demand, but it essentially includes goldblotch groupers (*Epinephelus costae*, 70%), dusky grouper, *(Epinephelus marginatus*,15%), brown meagre (*Sciaena umbra*), common dentex (*Dentex dentex*), seabeams (*Diplodus* spp, *Sparus* spp), baraccudas (*Sphyraena barracuda*) and common octopus (*Octopus vulgaris*) with 15%.

**Table 2:** Effort and CPUE of the Spearfishing sector, used to estimate catches, 2011–2015.

Year	Number of licenses	Fishing days per year	Weight (kg)	Catch (tonnes)
2011	620	50	20	620
2012	696	50	20	696
2013	693	50	20	693
2014	743	50	20	743
2015	730	50	20	730

Overall, total recreational catches in Algeria increased from 6,149 tonnes in 2011 to 6,695 tonnes in 2015, with a peak of 7,600 tonnes in 2013 due to the peak of the number of active licenses.

### Revenues of the recreational fishing sector

The total revenue generated by recreational fisheries is estimated at around $41.8 million US per year, of which $40, 2 million US is landed value, if we remove operational costs ($2, 288 139 US), the net income is estimated at $39.58 million US, and around $1, 607 580 US is earnings that would otherwise be taxed by the government if the sector’s catches were accordingly regulated, hence constituting a loss to the government of Algeria in taxes. The total revenue per vessel is equivalent to $2, 400 US monthly, which is almost 6.1 times higher than Algerian average salary of 392 USD (http://www.tsa-algerie.com/20161119/algeriens-gagnent-moyenne-39-200-da-net-mois/). The landed value for spearfishers is assessed at $4.9 million US, or $562 US·month^-1^·spearfisher^-1^, which is 1.4 times higher than the Algerian average salary. Considering the illusion that catches are not sold, none of this income is captured by the Government of Algeria, and despite regulations–which otherwise lack implementation–most of this value is captured in the form of a nontaxable, black market income.

### Uncertainties with the results

The results assessed in this study appear to be very conservative for various reasons:

- The annual CPUE is an average landing of 9 boats of one ports among 32 port in Algeria and it is based on one sea trip per day while many small scale boat perform 2 to 3 fishing trips per day;
- The number of recreational authorization is decreasing, between 2016 and 2013 but the number of boat are increasing [22], highlighting that boats that are not registered are not included in this analysis;

## Discussion

The recreational reconstructed catch in Algeria reached 6,000 tonnes in 2015, which is higher comparing to previous estimates of annual recreational catches ranged between 380 t [23], and 1,000 t [9], The case of Boumerdes shows that, potentially, only 1% (7.5 t reported from 599 t estimated) of the total recreational catch is ever reported. This may be due to the fact that remote landing beaches and other improvised landing places, amounting a total of 32 along the Algerian coastline [24,25], are not accounted for in official statistics. However, given that our estimate is based on all licensed vessels, we believe that by extrapolating an average observed CPUE on the total authorized fleet, all authorized vessels are captured in our estimate. Sumaila [26] Classified three forms of subsidies; “Beneficial subsidies” or “good”, they enhance the growth of fish stocks through conservation, and the monitoring of catch rates through control and surveillance measures, “Capacity-enhancing subsidies” or “bad” which are all forms of capital inputs that reduce cost or enhance revenue,”Ambiguous subsidies” or “ugly” are defined as programs whose impacts are undetermined. We highlight, herein, that in addition to the catch under-reporting, both the regulation status and the reporting system are questionable. Overall, the fishery generates over 45 million USD in revenue that remains part of the informal economy, “given our findings, recreational fishers enjoy a discounted price for licences while they compete on the same market and for the same stocks of fish than artisanal fishers. Given the lack of oversight on the fishery, which translates into unreported catches and important losses to the government, we argue that the discount offered is a form of capacity enhancing subsidy, which is one of the bad forms of subsidies” which have, in addition to potential negative impacts on the environment, far reaching socio-economic impacts, when ill-managed [26]

The Algerian law clearly states that sports and recreational fishing products cannot be sold or exchanged, and are only intended for self-consumption or release. “*Recreational fishing practiced with recreational vessels is subject to an authorization delivered by the administration responsible for the fishing*” [15]. Respective of the conditions above, the difference in licensing fees amounting at a total of over $1, 607 580 US (1170 USD annually per fisher) as assessed in this study serves as an incentive for many artisanal fishers to convert to a recreational activity because of the lack of control in the latter, and lower post-harvest costs, overall. In fact, whereas the previous law precisely defined the conditions for the practice of recreational fishing (distance, gear and period restrictions) [27], the new regulations adopted as of 2011 do not provide any specificities with regards to potential infractions, limiting only the purpose of the catch that has to be for self-consumption and not for sale [10], In 2015, this law was modified and supplemented, and a fine is imposed to anyone who practice recreational fishing in contravention of the provisions of this law [28]. Without any further precisions, that means that the commercialization of catch remains the only infraction.

Thanks to this legal loophole, many recreational vessels operate commercially [20] and outcompete artisanal fishers. Indeed, the number of recreational licenses is 1.8 times higher than the number of artisanal (commercial) licenses, for the same vessel length range, and lower restrictions on the recreational sector [11, 29, 30]. The highest difference in number of licenses between the two sectors is observed in Tipaza, Chlef, Mostaganem and Oran, where controls and monitoring by the coast guards are severely lacking.

Another difference that highlights a lack of monitoring capacity occurs between the number of authorizations as reported by local departments of fisheries (2,067 recreational vessels licensed in 2013), and those reported by the Central Ministry of Fisheries (25,042 for the same year) [31], denoting of a major discrepancy between the central and regional governments.

Although spearfishing is closely regulated [10], restrictions on diver’s age, fishing times, and distance from shore are often infringed upon. In addition to authorized spearfishers not abiding by the law, a high number of unauthorized are operating along the coast, which means that the catch estimates and values presented herein are likely conservative.

This paper sheds some light on the far-reaching negative social and economic implications of the so-called recreational fisheries of Algeria, whose impacts on the government are often assumed positive when compared to commercial sectors. Indisputably, far from being for sports and recreational purposes, the economic value that these fisheries generate, while escaping local controls, and providing a tax shelter for fishers outcompeting traditional small-scale fishers facing declining fish stocks [9], still contribute somehow to social peace in the country through supplying human nutrition and food security [32]. The dynamics of this sector remain unique in the sense that there is a general attitude of *laissez faire* within the government management system–which would have to face yet again social frustration by the Youth—, Hence, these issues need to be urgently addressed given that catches are drastically declining across sectors [9]. The issue of competition between artisanal (traditional) and recreational sectors is reflected through other examples globally, yet it remains largely understudied. Indeed, Canadian Pacific fisheries whose management is based on a output controls generated an important dispute over access between the recreational fishing lobby and the traditional indigenous fishing communities when 12% of the overall fishing quota was reallocated to the recreational sector. This issue was exacerbated by the fact that the recreational sector's catch in Canada’s Pacific, when monitored, appears to exceed quota [33]. This allocations has alienated small-scale and indigenous fishing communities at various occasions and remain an ongoing issue, mainly driven by access priorities, and the lack of knowledge on the impacts of recreational fisheries [33, 34]. Similarly, the lack of knowledge on recreational fisheries in the Bahamas [35] creates a tremendous burden on the ecosystem,

This problem is also observed in Caribbean marine protected areas, in which fees of scuba diving collected, which could contribute to resources conservation cost, do not reflect the real number of divers neither the real ecological impact [36]. On a global scale, universal code of conduct for the practice and management of recreational fisheries is required [37]. In fact, even where recreational fishing sector is controlled, obeys to the leisure principle, and where fishes are released, there is an ecological impact and contribution on global fish stock decline [38–41]. This unsuspected additional pressure, on the marine resource in Algeria suggests a higher ecological impact. The imprecise regulation of recreational sector allows a "legitimate" confusion with commercial sector and the lack of control is "trivializing" a non conformity. These two points are the mains issues highlighted in this paper. Managing recreational fisheries, within the fragile social environment of Algeria, where subsidizing recreational fisheries is perceived as a levy to reduce social pressures [42–44] is a difficult but necessary task. Herein, we argue that as the footprint of recreational fisheries is higher than that shown in official figure, the losses both on stocks, commercial fishers, and the government are important, there is an urgent need for better monitoring of the fishery.

## Supporting information

S1 Table CPUE and number of fishing trip of the nine recreational vessels used in this study. (XLSX)
